# UHPLC/MS Profile and Antimalarial Potency of *Enantia chlorantha* Oliv. (Annonaceae) Stem Bark Aqueous Extract

**DOI:** 10.1155/adpp/5068693

**Published:** 2025-12-22

**Authors:** Abel Narcisse Messi Betene, Raceline Gounoue Kamkumo, Patrick Valère Tsouh Fokou, Florence Ngueguim Tsofack, Michel Arnaud Mbock, Eugenie Aimée Madiesse Kemgne, Albertine Ngako, Loic Steve Ngwem Tenlep, Marius Jaures Tsakem Nangap, Roberto Fokou, Darline Dize, Pascal Owona, Mariscal Brice Tchatat Tali, Fabrice Fekam Boyom, Théophile Dimo

**Affiliations:** ^1^ Department of Animal Biology and Physiology, University of Yaoundé I, Yaoundé, Cameroon, uy1.uninet.cm; ^2^ Department of Biochemistry, University of Yaounde I, Yaoundé, Cameroon, uy1.uninet.cm; ^3^ Department of Biochemistry, University of Bamenda, Bambili, Cameroon, unibda.net; ^4^ Department of Biochemistry, University of Douala, Douala, Cameroon, univ-douala.cm

**Keywords:** antimalarial activity, *Enantia chlorantha*, *plasmodium*, rats, UHPLC/MS

## Abstract

The escalating challenge of malaria management, primarily driven by antimalarial drug resistance, necessitates the urgent exploration of novel therapeutic agents. This investigation focused on characterizing the therapeutic efficacy of an aqueous stem bark extract derived from *Enantia chlorantha* Oliv (Annonaceae) against the parasitic burden of *Plasmodium berghei* in a rodent model. Comprehensive assessment revealed noteworthy *in vitro* antiplasmodial efficacy against both the *PfDd2* and *Pf3D7* strains of *P. falciparum*, evidenced by median inhibitory concentrations (IC50) of 1.002 and 19.040 μg/mL, respectively. Moreover, the extract demonstrated a statistically significant, dose–responsive suppression of parasitemia in the *in vivo* model (*p* < 0.001), achieving suppression rates between 79.00% and 96.91%. Critically, the administration of the extract mitigated malaria‐associated pathophysiology, including the prevention of cachexia, anemia, and elevated leukocyte counts. It concurrently facilitated the functional recovery of hepatic and renal biomarkers (e.g., transaminases, bilirubin, and creatinine), reversed indicators of cellular oxidative stress, and potentially lessened multiorgan structural damage. Collectively, these preclinical findings robustly support the substantial antimalarial capacity of *E. chlorantha* stem bark extract, providing scientific validation for its ethnobotanical application. Future pharmacological research is now imperative to isolate and chemically identify the specific phytochemical constituents responsible for these observed bioactivities.

## 1. Introduction

Malaria remains a major global health threat, particularly in tropical and subtropical regions, with a disproportionately high impact on sub‐Saharan Africa. Recent data indicate that this region accounts for 95% of malaria cases in Africa, resulting in 619,000 deaths, predominantly among children under five and pregnant women [1]. The systemic nature of *Plasmodium* infection can lead to multi‐organ damage and a range of clinical symptoms, including fever, severe anemia, thrombocytopenia, and various neurological and respiratory complications [2]. These pathophysiological changes are often linked to impaired microcirculation caused by the sequestration of parasitized red blood cells (RBCs), as well as systemic inflammatory responses and endothelial activation [2]. Furthermore, *Plasmodium* parasites require lipids for their growth and replication, relying on a Prokaryotic Fatty Acid Synthesis Type II (FAS II) pathway located in the apicoplast organelle, which is crucial for their replication during the pathogenic blood stage [3]. Although significant progress in malaria treatment has been made with plant‐derived compounds like quinine (from *Cinchona officinalis*) and artemisinin (from *Artemisia annua*), the emergence of parasite resistance to these and other available drugs has hindered global eradication efforts. This growing resistance has created an urgent need for the discovery of alternative and complementary treatments. The sub‐Saharan African region, which bears the heaviest disease burden, is also home to a vast array of medicinal plants used for various health conditions [4]. Numerous studies have focused on identifying new antimalarial agents from natural sources [5, 6]. Among these, *Enantia chlorantha* Oliv. (Annonaceae), also known as *Annickia chlorantha*, is a plant traditionally used to treat malaria, jaundice, urinary tract infections, hypoglycemia, typhoid fever, etc. [7]. Previous research has identified various bioactive compounds such as alkaloids, flavonoids, and tannins, which are responsible for its antimicrobial, antiviral, antimalarial, and antipyretic properties [8]. Given these reports, the objective of this study was to evaluate the *in vitro* and *in vivo* antiplasmodial activity of the aqueous extract of the stem bark of *E. chlorantha*.

## 2. Materials and Methods

### 2.1. Plant Material and Extraction Procedure

The stem bark of *E. chlorantha* (Annonaceae) was collected in Andok, Mefou‐et‐Akono Division, Centre Region in Cameroon. The authentication of the material was conducted at the National Herbarium of Cameroon (HNC) against a voucher specimen (Ref. No.: 32065/SRF/Cam). Following collection, the *E. chlorantha* stem bark was coarsely chopped, dried at ambient temperature (25 ± 2°C), and underwent subsequent pulverization. A 500 g of portion of the resulting powder was macerated in 5 L of distilled water for 24 h under continuous agitation. Filtration was achieved using Whatman filter paper (No. 4), yielding an aqueous filtrate, which underwent desiccation in an oven at a temperature of 45°C. The final crude powdered extract was weighed and then stored in a sealed container for subsequent experimental utilization.

### 2.2. Animal Material

The study used female nulliparous Wistar rats aged between eight and nine weeks, with an approximate body weight of 150 ± 10 g. Selecting nulliparous animals of a consistent age and weight minimizes physiological variations across the cohort, thereby providing clearer insights into the pharmacological effects of the extract. Animals were sourced, bred, and then acclimatized in the animal facility at the Faculty of Science, University of Yaoundé I, Cameroon. Housing conditions involved ambient temperature conditions (25 ± 2°C), a natural 12‐h light–dark cycle, and *ad libitum* provision of both drinking water and standard laboratory feed. All experimental procedures were conducted in strict accordance with ethical guidelines, specifically authorized by the Cameroon National Ethical Committee (Ref. No.: FW‐IRB00001954).

### 2.3. Phytochemical Analysis of the Extract

#### 2.3.1. Determination of Alkaloid Content

The total alkaloid content of the plant extract was determined using the method described in [9], with minor modifications. Briefly, the extract (100 mg) was dissolved in 10 mL of 80% ethanol solution and then homogenized and centrifuged at 500 g for 10 min. One milliliter of the supernatant, one mL of acidified FeCl_3_ solution (0.025 M, 0.5 M HCl), and one milliliter of an ethanol solution of 1,10‐phenanthroline (0.05 M) were introduced into a test tube, and the mixture was incubated for 30 min at 100°C in a water bath. The absorbance of the reddish complex formed was read at 510 nm against the blank. Quinine (10 μg/mL) was used as the standard. The total alkaloid content was determined from the calibration curve (*R* ^2^ = 0.99) and expressed as micrograms of quinine equivalent per gram of dry matter (μg QiE/g DM).

#### 2.3.2. Determination of Total Polyphenol Content

Total polyphenol content was determined using the Folin–Ciocalteu method, as described by Mbopi et al. [10]. To 100 μL of the extract solution, 500 μL of Folin–Ciocalteu reagent (diluted tenfold) was added. Two minutes later, 2 mL of 20% sodium carbonate was added, and the mixture was thoroughly mixed. After incubating for 30 min at room temperature in the dark, the absorbance of the mixture was read at 760 nm against the reagent blank. Each assay was performed three times for each sample concentration. Polyphenol quantification was carried out on the basis of a linear calibration curve, generated using a tannic acid standard at different concentrations ranging from 25 to 125 μg/mL, under the same conditions as the sample. The results were expressed as micrograms of tannic acid equivalent (TAE) per gram of dry matter (μg TAE/g DM).

#### 2.3.3. Determination of Total Flavonoid Content

The colorimetric method was employed to determine the total flavonoid content as described by Jampou et al. [11]. 500 μL of the extract was added to 1.5 mL of methanol. Then, 100 μL of 10% aluminum chloride, 100 μL of 1 M sodium acetate, and 2.8 mL of distilled water were added. The mixture was incubated at room temperature for 30 min, and the absorbance was read at 415 nm against the reagent blank. The experiment was performed in triplicate for each product concentration tested. A calibration curve was constructed using serial dilutions of quercetin ranging from 60 to 300 μg/mL. The total flavonoid content was then calculated from the calibration curve and expressed as micrograms of quercetin equivalent per gram of dry matter (μg QE/g DM).

#### 2.3.4. Determination of Tannin Content

The method described in Ref. [12] was used to estimate the tannin content of the plant extract. One milliliter of the extract was mixed with 5 mL of working solution (50 mg vanillin + four mL of 1 N HCl in 100 mL distilled water), and the mixture was incubated at 30°C for 20 min. The absorbance was then read at 500 nm against the blank. Tannic acid (500–1.000 μg/mL) was used as a standard, and the resulting calibration curve was used to calculate the tannin content of the extract. The tannin content was then calculated and expressed as micrograms of TAE per gram of dry matter (μg TAE/g DM).

#### 2.3.5. UHPLC‐MS Analysis of *E. chlorantha* Aqueous Extract

Ultrahigh resolution mass spectra were acquired using a quadrupole time‐of‐flight (QTOF) spectrometer (Bruker, Germany), which was coupled with a HESI source, following previously established methodology by Tsakem et al. [13]. The instrument was operated in the negative ion mode, scanning a mass range between 100 and 1500 *m*/*z* at a 1.00 Hz frequency. Automatic gain control was engaged to guarantee highly accurate mass measurements, achieving a deviation of less than 0.40 ppm using Na Formate as the calibration standard. Specific parameters included a 3.5 kV spray voltage and a capillary temperature set at 200°C. Nitrogen gas was employed as the sheath gas at a flow rate of 10 L/min. The spectrometer was interfaced with an Ultimate 3000 (Thermo Fisher, USA) UHPLC system, which integrated a solvent pump, a diode array detector (DAD) spanning 190–600 nm, an autosampler (5 μL injection volume), and a column oven fixed at 35°C. Chromatographic separations were achieved using a Synergi MAX‐RP 100A (50 × 2 mm, 2.5 μ particle size). The mobile phase involved an aqueous solvent (A: H_2_O with 0.1% HCOOH) and an organic solvent (B: acetonitrile with 0.1% HCOOH), run at a constant flow rate of 500 μL/min. A specific elution gradient was implemented: 95% A was maintained isocratically for the initial 1.5 min; this transitioned to 100% B through a linear gradient over the subsequent over 6 min; 100% B was held for 2 min; and the system was then returned to the starting composition (90% A) within 1 min and re‐equilibrated for 1 min. Putative peak identification relied upon comparing the peaks in the determined compound masses and fragmentation data with reported values available in the SciFinder database.

### 2.4. Evaluation of the *In Vitro* Antiplasmodial Activity of the Extract

The *in vitro* antiplasmodial activity of the extract was evaluated on *Plasmodium falciparum* chloroquine sensitive (*Pf3D7*) and multidrug resistant (*PfDd2*), which were provided by Bei Resources (USA). The methods for cultivating the parasites [14] and for performing the drug susceptibility test [15] were carried out as previously described by Tsakem et al. [13]. In brief, parasite strains were cultivated in sealed flasks in the RPMI 1640 medium supplemented with 10% heat‐inactivated human serum and human erythrocytes to achieve a hematocrit of 2%. The parasites were synchronized to the ring stage using a 5% sorbitol treatment. The plant extract was prepared at a stock concentration of 1 mg/mL in DMSO, while artemisinin and chloroquine were used as the reference drugs. The drug susceptibility test utilized the SYBR Green I–based fluorescence method. Fluorescence was measured using a Tecan Infinite M200 multiwell plate reader with excitation and emission wavelengths centered at 485 nm and 538 nm, respectively. The concentration that inhibits 50% inhibition of parasite growth (IC50) was determined using the dose–response curve (GraphPad Prism 8.01).

### 2.5. Cytotoxicity Assay of the Plant Extract

To evaluate the selective toxicity of the plant material, its *in vitro* cytotoxic properties were determined using human foreskin fibroblast (HFF) cells. These cells were maintained in a complete medium formulated with 13.5 g/L DMEM, 10% fetal bovine serum, 0.2% sodium bicarbonate (w/v) (Sigma), and 50 μg/mL gentamicin [16]. Cells were initially seeded into 96‐well, flat‐bottom tissue culture plates seeded at a density of 10^4^ cells/mL/well. After 24 h of incubation, 80 μL of each test solution was introduced to the wells. The extract was assayed across a concentration range from 0.24 to 125 μg/mL. The negative control received 0.4% DMSO (v/v) only. The plates were subsequently incubated for 48 h in a humidified atmosphere at 37°C with 5% CO_2_. Cell viability was quantified using the MTS/PMS kit solution (Promega). A volume of 20 μL of kit reagent was dispensed into each well, gently homogenized, and incubated for 3 h at 37°C. Following the removal of the overlying culture medium, 100 μL of the quenching solvent (0.4% DMSO) was added to the cellular pellets. The resultant formazan formation rate was then assessed by measuring the optical density (OD) at 490 nm using a microtiter plate reader (BioTek EL800, USA). Mean ODs were plotted against the corresponding drug concentrations. The 50% cytotoxic concentration (CC50) value was calculated using GraphPad Prism 8.01 software. Finally, the selectivity index (SI) against *P. falciparum* was determined by comparing the ratio of the HFF cell toxicity (CC50) to the IC50 (antiplasmodial activity),

(1) SI=CC50HFF cytotoxicityIC50Antiplasmodial activity.

### 2.6. *In Vivo* Evaluation of the Antimalarial Activity of *E. chlorantha*

The experiment was conducted using *P. berghei* (NK‐65) for the rat infection. The *in vivo* antiplasmodial assay was performed following the standardized protocol previously described by Kamkumo et al.​ with minor modifications [17]. In brief, rats were inoculated intraperitoneally with blood containing 1 × 10^6^ parasitized RBCs. Infected animals were then randomized and orally treated for five days with either distilled water, chloroquine sulfate, or the aqueous extract of *E. chlorantha* at doses of 125, 250, and 500 mg/kg. Parasitemia and body weight were monitored daily throughout the experimental period. Parasitemia was assessed by examining Giemsa‐stained thin blood smears under a microscope at 100× magnification with oil immersion, and the percentage was calculated using the following formula: Parasitemia (%) = 100 (Number of parasitized erythrocytes/Total number of erythrocytes) [18].

The inhibitory activity of the extract was determined 24 h after the final treatment using the following formula: Inhibition% = 100 [(Parasitemia in malaria control − Parasitemia at the given dose)/Parasitemia in malaria control].

#### 2.6.1. Sample Collection and Analysis of Some Physiological Parameters

At the end of the study period, the experimental animals were sacrificed under anesthesia with the combination of diazepam (30 mg/kg) and ketamine (10 mg/kg). Whole blood was collected into EDTA tubes for hematological profiling. A separate quantity of blood was collected into dry tubes and subjected to centrifugation at 500*g* for 15 min at 4°C. The resulting supernatant (serum) was carefully collected and used for biochemical analysis. Critical visceral organs, specifically the liver, kidney, spleen, and brain, were quickly excised and documented for weight. A precise fraction of each organ—0.4 g of liver, 0.2 g of kidney, 0.4 g of spleen, and 0.2 g of brain—was allocated for the quantitative measurement of oxidative stress markers. The remaining tissues from each organ were preserved via immersion in 10% buffered formalin solution for preparation toward histopathological examination.

#### 2.6.2. Hematological Parameter Assessment

The quantification of various hematological parameters was achieved using a Sysmex XP‐300 automated hematology analyzer (Germany). The panel of blood parameters examined included the RBC count, hemoglobin​ (Hb) level, packed cell volume (HCT), white blood cell (WBC) count, and the circulating platelet (PLT) concentration.

#### 2.6.3. Evaluation of Some Biochemical Parameters

The serum samples were subjected to the quantitative analysis for several key biomarkers. Assays for aspartate aminotransferase (AST) and alanine aminotransferase (ALT) activities, total bilirubin, total proteins, and creatinine levels using commercial diagnostic kits (Biolabo, France). The resulting sample absorbance values were measured and recorded with a UVLine S/5000 spectrometer (SI analytics®, Germany). For the assessment of oxidative stress markers, designated organ sections were initially homogenized in 2 mL of 50 mM Tris‐HCl buffer (pH 7.4). The resulting homogenate was then subjected to centrifugation at 1500*g* for 25 min at a temperature of 4°C. The clear supernatant obtained was reserved for the measurement of several indicators: malondialdehyde (MDA), nitrite (NO) and reduced glutathione (GSH) concentrations. Additionally, the enzymatic activities of catalase and SOD were determined, all following established, standardized protocols [19–22].

#### 2.6.4. Histopathological Analysis

Histopathological analysis of organs was performed using the conventional hematoxylin–eosin (HE) staining method, as previously described by Kamkumo and coworkers [23]. Serial paraffin sections at 5 μm were stained with HE for examination under a light microscope using an Olympus 20×/100× objective (HE × 200) and captured with a microscopic camera (ZEISS Axioscope, Germany).

### 2.7. Statistical Analysis

Data were expressed as mean ± standard error of the mean (SEM). Statistical analysis of results was conducted using ANOVA followed by Tukey’s post hoc test with GraphPad Prism software, Version 8.01. A *p* value < 0.05 was considered statistically significant.

## 3. Results

### 3.1. Biometabolites Contents and LC/MS Chemical Profile of the Extract

**Figure 1:**
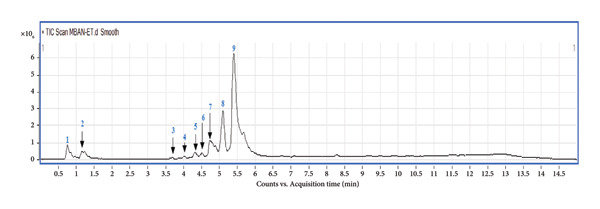
UHPLC‐MS profile of aqueous leaves extract of the *E. chlorantha* Oliv stem bark aqueous extract.

**Figure 2:**
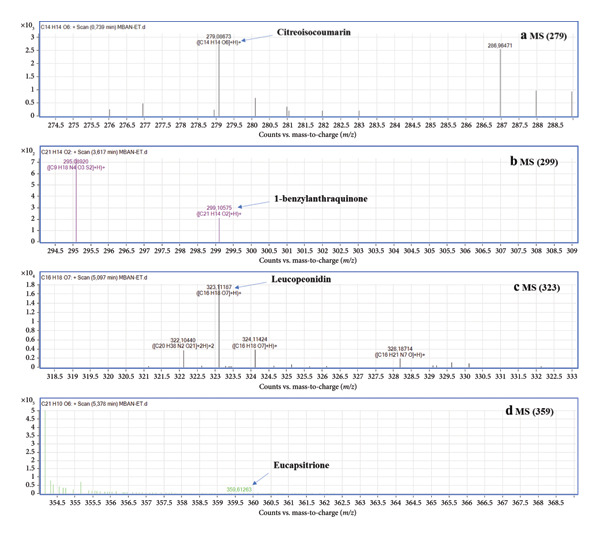
MS spectra of Compounds 1 at *m*/*z* 279 (a), 3 at *m*/*z* 299 (b), 8 at *m*/*z* 323 (c), and 9 at *m*/*z* 359 (d) in the positive ion mode.

The extract appears to be richest in polyphenols, followed by alkaloids, flavonoids, and then tannins (Table 1). The significant presence of polyphenols in the extract warrants further investigation due to their potential antimalarial properties documented in various studies [24]. Furthermore, the presence of alkaloids, flavonoids, and tannins also holds promise. These classes of compounds are recognized for their diverse biological activities, including antimicrobial and anti‐inflammatory properties [25]. Their presence alongside polyphenols suggests a potentially multifaceted pharmacological profile for the extract, potentially contributing to its overall antimalarial effect. Furthermore, UHPLC‐MS analysis revealed nine major peaks in the chemical profile of the extract (Figure 1 and Table 2) among which four compounds were identified (Figure 2 and Table 2).

**Table 1:** Quantitative biometabolite contents in the extract.

Polyphenols (μg TAE/g extract)	Alkaloids (μg QiE/g extract)	Flavonoids (μg QE/g extract)	Tannins (μg TAE/g extract)
1496.22 ± 972.29	254.60 ± 33.40	195.40 ± 4.80	97.80 ± 9.49

Abbreviations: QE = quercetin equivalent, QiE = quinine equivalent, TAE = tannic acid equivalent.

**Table 2:** Main signals exhibited in the LC‐MS spectra of compounds detected in *E. chlorantha* Oliv trunk bark aqueous extract and proposed attribution.

No	Tr (min)	[*M* + *H*]+		Err (ppm)	Molecular formula	Name of compound
		Exp.	Calcl.			
1	0.739	279.0867	278.0790	−3.30	C_14_H_14_O_6_	Citreoisocoumarin
2	1.211	269.1164	268.1099	2.81	C_17_H_16_O_3_	Not identified
3	3.617	299.1057	298.0993	3.04	C_21_H_14_O_2_	1‐Benzylanthraquinone
4	4.014	285.1483	284.1412	0.75	C_18_H_20_O_3_	Not identified
5	4.320	357.1857	356.1776	−2.53	C_25_H_24_O_2_	Not identified
6	4.485	311.1264	310.1205	4.23	C_19_H_18_O_4_	Not identified
7	4.741	359.0397	358.0324	0.08	C_17_H_10_O_9_	Not identified
8	5.097	323.1118	322.1052	1.76	C_16_H_18_O_7_	Leucopeonidin
9	5.378	359.0567	358.0477	4.9	C_21_H_10_O_6_	Eucapsitrione

### 3.2. *In Vitro* Antiplasmodial Activity of the *E. chlorantha* Aqueous Extract

Table 3 presents the *in vitro* antiplasmodial activity of the *E. chlorantha* aqueous extract against *P. falciparum* strains. The extract displayed moderate‐to‐good inhibitory effects on parasite growth, with *PfDd2* being more susceptible than *Pf3D7*. Notably, the extract exhibited no cytotoxicity toward HFF Vero cells up to a concentration of 100 μg/mL.

**Table 3:** *In vitro* antiplasmodial and cytotoxicity effects of the extract.

Substances	Parasite strains	IC_50_ ± SD	CC_50_	SI
*Enantia chlorantha* (μg/mL)	*P*. *f*. Dd2	1.00 ± 0.19	> 100	100
	*P*. *f*. 3D7	19.04 ± 0.42	> 100	5

Chloroquine sulfate (μM)	*P*. *f*. Dd2	0.827 ± 0.122	—	—
	*P*. *f*. 3D7	0.031 ± 0.004	—	—

Artemisinin (μM)	*P*. *f*. Dd2	0.037 ± 0.001	—	—
	*P*. *f*. 3D7	0.032 ± 0.001	—	—

*Note:* Each value represents the mean ± SD.

Abbreviations: CC_50_ = cell cytotoxicity of the extract on normal human foreskin fibroblast (HFF) cells, IC_50_ = median inhibitory concentration, SD = standard deviation, SI = selectivity index.

### 3.3. Effects of the Extract on the Parasitemia of Infected Animals

**Figure 3:**
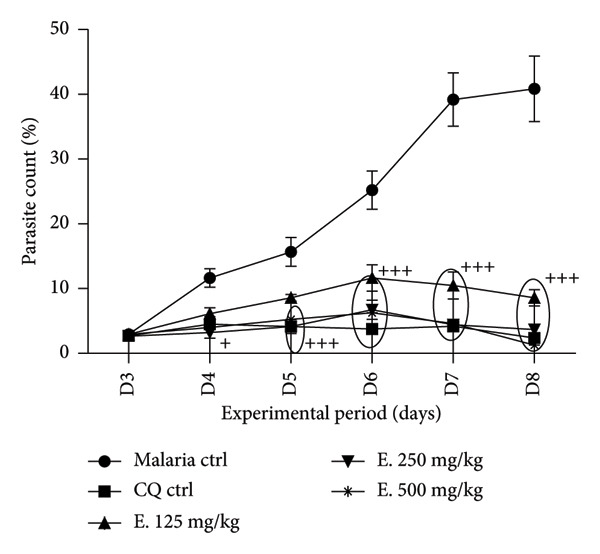
Effects of the *Enantia chlorantha* aqueous extract on parasitemia in the *Plasmodium berghei*–infected rat. Each point represents the mean ± SD, *n* = 5, ^+^ *p* < 0.05, ^+++^ *p* < 0.001: significant difference compared to malaria control. Malaria ctrl = *P. berghei*–infected rat treated with distilled water (10 mL/kg); CQ ctrl = Infected rat receiving chloroquine (10 mg/kg); E. 125, 250, and 500 mg/kg = infected rat treated with *E. chlorantha* extract at the respective doses of 125, 250, and 500 mg/kg.

The intraperitoneal inoculation of healthy animals with 500 μL of rat blood containing 1 × 10^6^ *P. berghei*–parasitized RBCs led to a parasite count of 43.77% in the malaria control group, after 8 days without treatment (Figure 3). The intake of *E. chlorantha* extract significantly reduced (*p* < 0.001) parasite growth in dose‐dependent manner, with inhibition percentages of 79.00%, 91.04%, and 96.91% at the respective doses of 125, 250, and 500 mg/kg, compared to malaria control. Notably, chloroquine treatment achieved a 94.15% inhibition rate.

### 3.4. Effects of *E. chlorantha* Aqueous Extract on the Body Weight of Infected Animals

**Figure 4:**
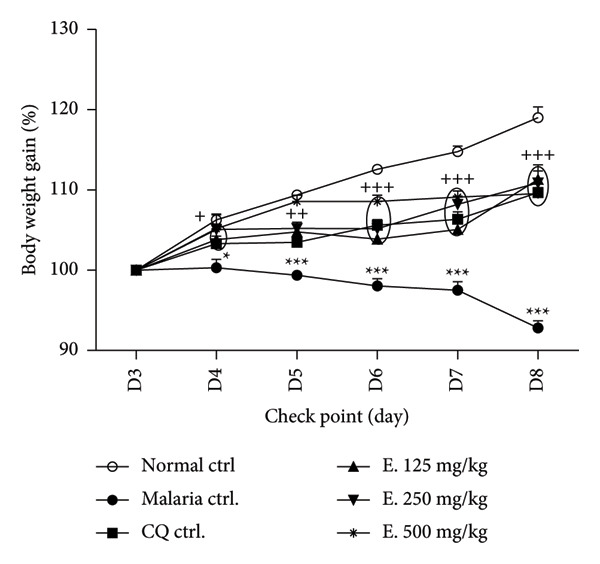
Effects of *E. chlorantha* aqueous extract on the body weight gain in *P. berghei–*infected animals. Points represent the mean ± ESM, *n* = 5, ^∗^ *p* < 0.05, ^∗∗∗^ *p* < 0.001: significant difference compared to normal control (Normal ctrl); ^+^ *p* < 0.05, ^++^ *p* < 0.01, ^+++^ *p* < 0.001: difference to malaria control; Malaria ctrl = infected rat treated with distilled water (10 mL/kg); CQ ctrl = Infected rat treated with chloroquine (10 mg/kg); E. 125, 250, and 500 mg/kg = infected rat and treated with *E. chlorantha* extract at the respective doses of 125, 250, and 500 mg/kg.

Figure 4 depicts the impact of *E. chlorantha* extract on the body weight of *P. Berghei*–infected animals. Malaria infection caused a progressive and significant (*p* < 0.001) decrease in body weight in the infected animals compared to the normal control group. This decline started at Day 4 by 5.97% (*p* < 0.01) and reached to 26.22% (*p* < 0.001) by Day 8. Conversely, daily administration of the *E. chlorantha* aqueous extract significantly (*p* < 0.001) increased body weight gain. Animals treated with 125, 250, and 500 mg/kg of the extract exhibited body weight gains of 18.46%, 18.08%, and 16.88%, respectively, compared to the malaria control group. Importantly, chloroquine treatment (10 mg/kg) also resulted in a significant body weight increase (*p* < 0.001) compared to the malaria control.

### 3.5. Effects of the Extract on Some Hematological Parameters

Malaria infection caused significant alterations in the blood parameters of infected animals compared to the normal control group (Table 4). RBCs count, hemoglobin (HGB) level, and hematocrit (HCT) rate decreased significantly by 64.75% (*p* < 0.001), 39.89% (*p* < 0.05), and 22.75% (*p* < 0.001), respectively, in the malaria control group. In contrast, malaria infection led to a significant increase in the WBC count by 190.54% (*p* < 0.001) and PLT count by 149.99% (*p* < 0.001). Treatment with the *E. chlorantha* aqueous extract significantly improved these blood parameters when compared to the malaria control group. All three doses (125, 250, and 500 mg/kg) significantly increased the RBC count (*p* < 0.05 or *p* < 0.01), HGB concentration (*p* < 0.01 or *p* < 0.001), and HCT rate (*p* < 0.001) with regard to the malaria control. The extract also significantly reduced the WBC count (*p* < 0.001) and PLT count. Similarly to the extract, chloroquine treatment significantly (*p* < 0.01 or 0.001) increased the RBC count, HGB level, and HCT rate, associated with a decrease in the WBC and PLT count compared to the malaria control group.

**Table 4:** Effects of the *Enantia chlorantha* aqueous extract on some hematological parameters of malaria‐infected rats.

Parameters	Nor ctrl	Mal ctrl	CQ ctrl	E. 125	E. 250	E. 500
RBC (10^3^/μL)	6.27 ± 0.87	2.21 ± 0.05^∗∗∗^	5.15 ± 0.02^++^	4.40 ± 0.04^+^	5.40 ± 0.12^++^	4.76 ± 0.14^++^
HGB (g/dL)	13.06 ± 0.18	7.85 ± 0.11^∗^	14.20 ± 0.17^++^	15.15 ± 0.42^+++^	14.86 ± 0.16^++^	15.81 ± 0.31^+++^
HCT (%)	39.16 ± 1.11	30.25 ± 0.11^∗∗∗^	41.90 ± 0.74^+++^	42.00 ± 0.49^+++^	47.86 ± 0.35^+++^	47.95 ± 0.11^+++^
PLT (10^3^/μL)	352.33 ± 19.12	880.50 ± 9.16^∗∗∗^	422.66 ± 14.07^++^	506.50 ± 1.56^+^	612.50 ± 6.03^+^	418.50 ± 80.7^+++^
WBC (10^3^/μL)	7.40 ± 0.08	21.50 ± 1.16^∗∗∗^	7.13 ± 0.42^+++^	9.35 ± 0.42^+++^	11.10 ± 0.08^+++^	7.55 ± 0.11^+++^

*Note:* Values represent the mean ± ESM, *n* = 5. Nor crtl = healthy rats. Mal ctrl = malaria control = infected rat treated with distilled water (10 mL/kg); CQ ctrl = Infected rat treated with chloroquine (10 mg/kg); E. 125, E. 250, and E. 500 = infected rats and treated with *E. chlorantha* extract at the respective doses of 125, 250, and 500 mg/kg.

^∗^ *p* < 0.05 and ^∗∗∗^ *p* < 0.001: significant difference compared to normal control (Normal ctrl). ^+^ *p* < 0.05, ^++^ *p* < 0.01, and ^+++^ *p* < 0.001: significant difference compared to malaria control (Mal crtl).

### 3.6. Effects of the Aqueous Extract of *E. chlorantha* Stem Bark on Some Liver and Kidney Function Parameters

Malaria infection significantly altered liver and kidney function markers as recorded in Table 5. Infected animals exhibited elevated serum activities of ALT and AST, indicating liver function disturbance. Additionally, the bilirubin level increased, suggesting potential bile flow issues. Furthermore, the creatinine level, a marker of kidney function, was significantly higher in infected animals compared to the normal control. Conversely, the total protein concentration, a general indicator of overall health, significantly decreased in malarious rats. Treatment with the *E. chlorantha* aqueous extract for 5 days significantly improved these parameters when compared to the malaria control group. All extract doses (125, 250, and 500 mg/kg) significantly restored (*p* < 0.05 or *p* < 0.001) the levels of ALT, AST, bilirubin, and creatinine with regard to the malaria control. Additionally, all doses of the extract and chloroquine treatment significantly increased (*p* < 0.001) the total protein concentration compared to the malaria control group. Chloroquine treatment (10 mg/kg) also significantly reduced ALT, AST, creatinine, and bilirubin levels compared to the malaria control group.

**Table 5:** Effects of the aqueous extract of *E. chlorantha* on some liver and kidney parameters’ function in infected rats.

Parameters	Nor ctrl	Mal ctrl	CQ ctrl	E. 125 mg/kg	E. 250 mg/kg	E. 500 mg/kg
ALT (UI/L)	48.08 ± 0.85	89.40 ± 7.82^∗∗∗^	35.86 ± 0.46^+++^	38.47 ± 0.49^+++^	37.47 ± 1.23^+++^	36.97 ± 0.89^+++^
AST (UI/L)	130.76 ± 3.77	176.98 ± 6.14^∗∗∗^	129.20 ± 3.14^+++^	138.95 ± 1.32^+++^	126.42 ± 5.36^+++^	112.26 ± 3.60^+++^
Total bilirubin (mg/dL)	8.29 ± 0.54	10.16 ± 0.14^∗^	8.10 ± 1.93	8.36 ± 1.91	6.89 ± 1.77^+++^	7.11 ± 1.53^+++^
Total proteins level (mg/dL)	1.92 ± 0.11	1.10 ± 0.13^∗∗∗^	1.44 ± 0.31^+++^	1.41 ± 0.10^+++^	1.75 ± 0.21^+++^	1.66 ± 0.11^+++^
Creatinine (mg/dL)	5.17 ± 0.05	7.60 ± 0.34^∗∗∗^	4.92 ± 0.13^+++^	5.04 ± 0.06^+++^	5.04 ± 0.06^+++^	5.19 ± 0.10^+++^

*Note:* Each point represents the mean ± ESM, *n* = 5. Mal ctrl (malaria control) = infected rat treated with distilled water (10 mL/kg); CQ ctrl = infected rat treated with chloroquine (10 mg/kg); E. 125, 250, and 500 mg/kg = infected rat and treated with *E. chlorantha* extract at the respective doses of 125, 250, and 500 mg/kg.

^∗^ *p* < 0.05 and ^∗∗∗^ *p* < 0.001: significant difference compared to normal control (Normal ctrl); ^+^ *p* < 0.05 and ^+++^ *p* < 0.001: significant difference related to malaria control.

### 3.7. Effects of *E. chlorantha* Aqueous Extract on Some Parameters of Oxidative Stress

#### 3.7.1. Effects on the Malondialdehyde Level

**Figure 5:**
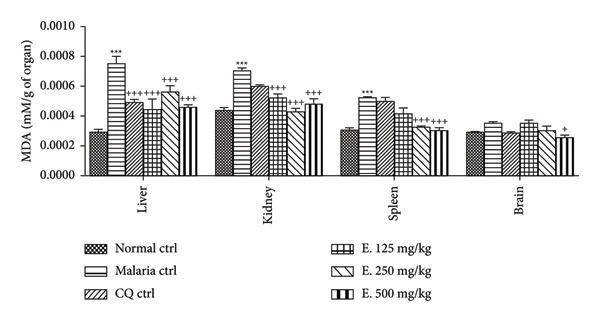
Effects of the *Enantia chlorantha* aqueous extract on the level of malondialdehyde (MDA) in some organs of malarious rats. Each point represents the mean ± ESM, *n* = 5; ^∗∗∗^ *p* < 0.001: significant difference compared to normal control (Nor. ctrl); ^+^ *p* < 0.05, ^++^ *p* < 0.01, and ^+++^ *p* < 0.001: difference compared to malaria control (Mal. Ctrl); Mal. ctrl = infected rat treated with distilled water (10 mL/kg); CQ ctrl = infected rat treated with chloroquine (10 mg/kg); E. 125, 250, and 500 mg/kg = infected rat and treated with *E. chlorantha* extract at the respective doses of 125, 250, and 500 mg/kg.

Figure 5 illustrates the impact of *E. chlorantha* extract treatment on some oxidative stress parameters during malaria infection. Compared to the normal control group, malaria significantly increased (*p* < 0.001) MDA levels in the liver by 60.95%, in the kidneys by 37.65%, and in the spleen by 41.24%. Daily administration of the *E. chlorantha* extract for 5 days significantly reduced (*p* < 0.001) the MDA level in the liver at all three doses (125, 250, and 500 mg/kg) by 40.84%, 25.19%, and 38.84%, respectively, compared to the malaria control group. Similarly, the extract significantly reduced MDA levels in the kidney (*p* < 0.001) by 25.77%, 38.92% and 31.73% at the respective doses of 125, 250, and 500 mg/kg and in the spleen (*p* < 0.001) by 37.71% and 41.90% at the respective doses of 250 and 500 mg/kg. Notably, only the highest extract dose (500 mg/kg) significantly reduced the MDA levels (*p* < 0.05) in the brain compared to the malaria control group. Chloroquine treatment (10 mg/kg) also significantly reduced the MDA levels in the liver (*p* < 0.001) compared to the malaria control group.

#### 3.7.2. Effects of *E. chlorantha* Aqueous Extract on the Nitrite Concentration

**Figure 6:**
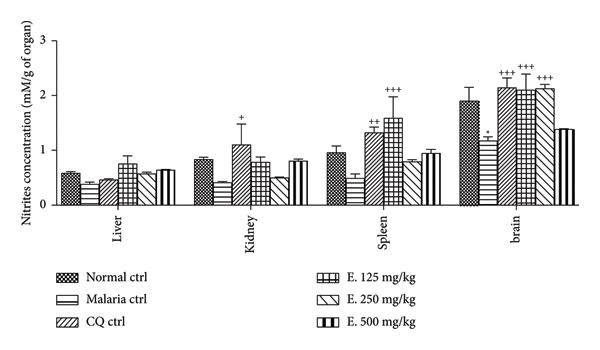
Effects of the *Enantia chlorantha* aqueous extract on the nitrite level of *Plasmodium* berghei–infected animals. Each point represents the mean ± ESM, *n* = 5; ^∗^ *p* < 0.05 and ^∗∗^ *p* < 0.01: difference compared to normal control (Nor. ctrl); ^+^ *p* < 0.05, ^++^ *p* < 0.01, and ^+++^ *p* < 0.001: difference compared to malaria control (Mal. Ctrl); Mal. ctrl = infected rat treated with distilled water (10 mL/kg); CQ ctrl = infected rat treated with chloroquine (10 mg/kg); E. 125, 250, and 500 mg/kg = infected rat and treated with *E. chlorantha* extract at the respective doses of 125, 250, and 500 mg/kg.

Figure 6 presents the effects of the *E. chlorantha* extract treatment on nitrite levels in malaria‐infected rats. *Plasmodium* infection caused a significant decrease (38.42%; *p* < 0.05) in brain nitrite levels compared to healthy animals. Daily administration of the *E. chlorantha* aqueous extract significantly increased (*p* < 0.001) nitrite levels in the brain. Compared to the malaria control group, the extract treatment resulted in 79.48% and 82.05% increase in brain nitrite levels at doses of 125 mg/kg and 250 mg/kg, respectively. Interestingly, the 125 mg/kg dose also significantly increased (*p* < 0.001) nitrite levels in the spleen by 1.69 times compared to the malaria control group. Chloroquine treatment (10 mg/kg) also significantly increased nitrite levels in the brain (by 82.90%; *p* < 0.001) and kidneys (by 1.68 times; *p* < 0.001) compared to the malaria control group.

#### 3.7.3. Effects of the Aqueous Extract of *E. chlorantha* on the Glutathione Rate

**Figure 7:**
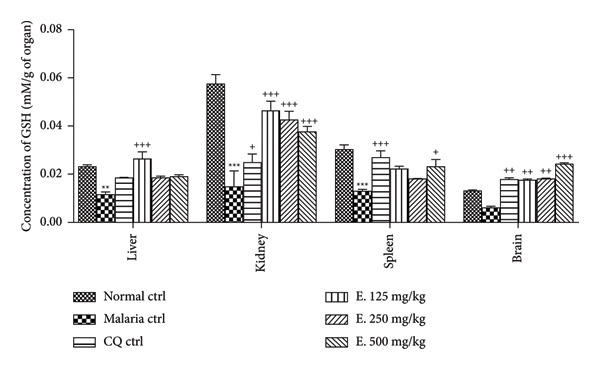
Effects of the *Enantia chlorantha* aqueous extract on reduced glutathione (GSH) in some organs of infected animals. Each point represents the mean ± ESM, *n* = 5; ^∗∗^ *p* < 0.01 and ^∗∗∗^ *p* < 0.001: significant difference compared to normal control (Nor. ctrl); ^+^ *p* < 0.05, ^++^ *p* < 0.01, and ^+++^ *p* < 0.001: difference compared to malaria control (Mal. Ctrl); Mal. ctrl = infected rat treated with distilled water (10 mL/kg); CQ ctrl = infected rat treated with chloroquine (10 mg/kg); E. 125, 250, and 500 mg/kg = infected rat and treated with *E. chlorantha* extract at the respective doses of 125, 250, and 500 mg/kg.

Figure 7 shows the effects of the *E. chlorantha* extract treatment on reduced glutathione (GSH) levels in malarious rat. Compared to the normal control group, malaria infection caused a significant reduction (*p* < 0.01) in the GSH levels in the liver by 50.00%, in the kidneys by 74.13% (*p* < 0.001), and in the spleen by 56.90% (*p* < 0.001). Daily administration of the *E. chlorantha* extract significantly increased (*p* < 0.001) the GSH levels across all examined organs. In the liver, the extract dose of 125 mg/kg significantly increased (*p* < 0.001) the GSH levels compared to the malaria control group. All three doses of the extract significantly boosted (*p* < 0.001) the GSH levels in the kidneys by 211.40%, 185.90%, and 153.02% at the respective doses of 125, 250, and 500 mg/kg. Likewise, a significant increase in the GSH concentration was recorded in the spleen at the dose of 500 mg/kg. Notably, the extract also significantly increased GSH levels in the brain at all doses of 1.83 (*p* < 0.01), 1.93 (*p* < 0.01), and 2.91 (*p* < 0.001) times compared to the malaria control group. Chloroquine treatment (10 mg/kg) significantly increased GSH levels in the kidneys (by 67.78%; *p* < 0.05), spleen (by 106.87%; *p* < 0.001), and brain (by 188.70%; *p* < 0.01) compared to the malaria control group.

#### 3.7.4. Effects of *E. chlorantha* Aqueous Extract on the Activity of Superoxide Dismutase (SOD)

**Figure 8:**
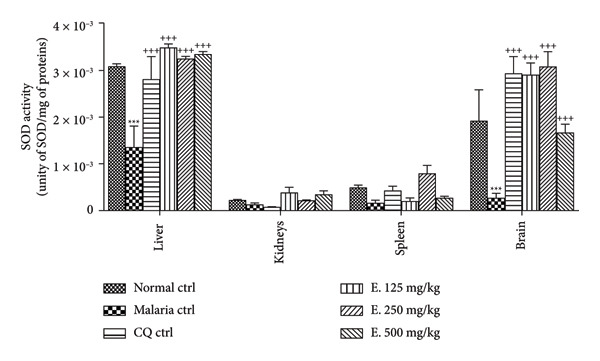
Effects of *Enantia chlorantha* aqueous extract on superoxide dismutase (SOD) activity in some organs of infected animals. Each point represents the mean ± ESM, *n* = 5; ^∗∗∗^ *p* < 0.001: significant difference compared to normal control (Nor. ctrl); ^+++^ *p* < 0.001: difference compared to malaria control (Mal. Ctrl. Mal. ctrl = infected rat treated with distilled water (10 mL/kg); CQ ctrl = infected rat treated with chloroquine (10 mg/kg); E. 125, 250, and 500 mg/kg = infected rat and treated with *E. chlorantha* extract at the respective doses of 125, 250, and 500 mg/kg.

Figure 8 summarizes the effects of malaria infection and *E. chlorantha* extract treatment on SOD activity in malarious animal. Compared to the normal control, *Plasmodium* infection caused a significant decrease in SOD activity in both organs; 56.66% decrease (*p* < 0.001) in the liver and a more substantial decrease of 89.47% (*p* < 0.001) in the brain. Treatment with the *E. chlorantha* extract significantly increased (*p* < 0.001) SOD activity in the liver and brain. Compared to the malaria control group, all three extract doses (125, 250, and 500 mg/kg) significantly increased SOD activity in the liver. Notably, the extract treatment demonstrated a remarkable effect on brain SOD activity, with increases exceeding 14.8 and 8 times the activity observed in the malaria control group at the doses 125 mg/kg and 250 mg/kg, respectively. Chloroquine treatment (10 mg/kg) also significantly increased (*p* < 0.001) SOD activity in both the liver (by 1.06 times) and brain (by 14.5 times) compared to the malaria control group.

#### 3.7.5. Effects of *E. chlorantha* Aqueous Extract on the Activity of Catalase

**Figure 9:**
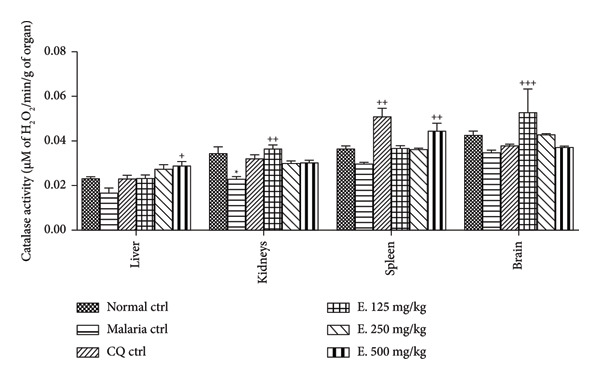
Effects of the *Enantia chlorantha* aqueous extract on the activity of catalase in some organs of *Plasmodium berghei*–infected animals. Each point represents the mean ± ESM, *n* = 5; ^∗^ *p* < 0.05: significant difference compared to normal control (Nor. ctrl); ^+^ *p* < 0.05, ^++^ *p* < 0.01, and ^+++^ *p* < 0.001: difference compared to malaria control (Mal. Ctrl. Mal. ctrl = infected rat treated with distilled water (10 mL/kg); CQ ctrl = infected rat treated with chloroquine (10 mg/kg); E. 125, 250, and 500 mg/kg = infected rat and treated with *E. chlorantha* extract at the respective doses of 125, 250, and 500 mg/kg.

Figure 9 illustrates the impact of the *E. chlorantha* aqueous extract on catalase activity in malaria‐infected animals. Compared to healthy animals, malaria infection caused a significant decrease (32.35%; *p* < 0.05) in catalase activity specifically within the kidneys. Treatment with the extract significantly increased (*p* < 0.01 or *p* < 0.05) catalase activity across some organs. At the lowest dose of the extract (125 mg/kg), a significant boost in catalase activity was noted as 56.52% (*p* < 0.01) in the kidneys and 52.94% in the brain related to malaria control. Furthermore, catalase activity significantly increased by 75.00% (*p* < 0.05) in the liver and 51.72% (*p* < 0.01) in the spleen of infected animals treated with the extract at 500 mg/kg.

### 3.8. Effects of *E. chlorantha* Aqueous Extract on the Weight and Histopathology of Some Organs in Infected Animals

#### 3.8.1. Effects of *E. chlorantha* Aqueous Extract on the Relative Weight of Some Organs

Effects of the *E. chlorantha* extract on the relative weight of organs in malaria‐infected rats are recorded in Table 6. Malaria infection significantly increased the relative weight of the liver (by 38.69%; *p* < 0.001), spleen (by 4.94 times; *p* < 0.001), and brain (by 21.27%; *p* < 0.05). Treatment with the *E. chlorantha* extract for 5 days significantly reduced (*p* < 0.05 or *p* < 0.001) the relative weight of the liver and spleen. Compared to the malaria control group, all three extract doses (125, 250, and 500 mg/kg) significantly decreased liver weight. The extract also significantly reduced spleen weight at all doses, with the highest dose (500 mg/kg) showing the most substantial decrease (83.18%; *p* < 0.001). Interestingly, only the 250 mg/kg dose of the extract significantly reduced brain weight (by 19.29%; *p* < 0.05) compared to the malaria control group. Chloroquine treatment (10 mg/kg) also significantly reduced (*p* < 0.001) the relative weight of the liver, spleen, and brain compared to the malaria control group.

**Table 6:** Effects of *E. chlorantha* aqueous extract on the relative weight of some target organs.

Organs	Nor ctrl	Mal ctrl	CQ ctrl	Ec 125 mg/kg	Ec 250 mg/kg	Ec 500 mg/kg
Liver	3.67 ± 0.07	5.09 ± 0.05^∗∗∗^	3.70 ± 0.04^+++^	4.45 ± 0.10^+^	4.05 ± 0.04^+++^	4.33 ± 0.02^++^
Kidneys	0.62 ± 0.03	0.60 ± 0.04	0.58 ± 0.01	0.57 ± 0.01	0.60 ± 0.01	0.63 ± 0.00
Spleen	0.57 ± 0.01	3.39 ± 0.08^∗∗∗^	0.61 ± 0.05^+++^	2.47 ± 0.13^+^	0.57 ± 0.02^+++^	1.39 ± 0.57^+++^
Brain	0.94 ± 0.02	1.14 ± 0.05^∗^	0.90 ± 0.01^+^	1.05 ± 0.01	0.92 ± 0.01^+^	1.05 ± 0.00

*Note:* Each point represents the mean ± ESM, *n* = 5. Malaria ctrl = infected rat treated with distilled water (10 mL/kg); CQ ctrl = infected rat treated with chloroquine (10 mg/kg); E. 125, 250, and 500 mg/kg = infected rat and treated with *E. chlorantha* extract at the respective doses of 125, 250, and 500 mg/kg.

^∗^ *p* < 0.05 and ^∗∗∗^ *p* < 0.001: difference compared to normal control (Nor ctrl). ^+^ *p* < 0.05, ^++^ *p* < 0.05, and ^+++^ *p* < 0.001: significant difference compared to malaria control.

#### 3.8.2. Effects of the Extract on the Microphotography of Some Organs

##### 3.8.2.1. Effect on the Liver

Figure 10 illustrates the effects of *P. berghei* infection and treatment on liver tissue. Panel A shows a healthy liver with well‐differentiated structures: portal vein (PV), bile canaliculus (BC), and hepatocytes (H). In contrast, Panel B shows the liver of a malaria‐infected animal (no treatment) with clear signs of damage: leukocyte infiltration (LI), dilated sinusoids (Scd), and swollen Kupffer cells (Kc) containing brown pigment (hemozoin, Hz). These alterations indicate liver inflammation and dysfunction. Treatment with the *E. chlorantha* extract at doses of 250 mg/kg (Panel D) and 500 mg/kg (Panel E) significantly improved liver health compared to the infected group (Panel B). Similarly, chloroquine treatment (10 mg/kg) in Panel C shows a restored liver architecture. These improvements suggest that the extract and chloroquine may have protective effects against liver damage caused by malaria infection.

Figure 10Effects of the extract of *E. chlorantha* stem bark on the microphotography of the liver section of *Plasmodium berghei*–infected rat (HE × 200). (a) Normal control; (b) malaria control; (c) chloroquine control; (d–f) test groups treated with the extract doses of 125, 250, and 500 mg/kg. Pv = Portal vein, He = hepatocyte, Kc = Kupffer cell, Bc = bile canaliculus, Ha = hepatic artery, Li = leukocyte infiltration, Scd = sinusoidal capillary dilatation, Hz = hemozoin.

**Figure:**
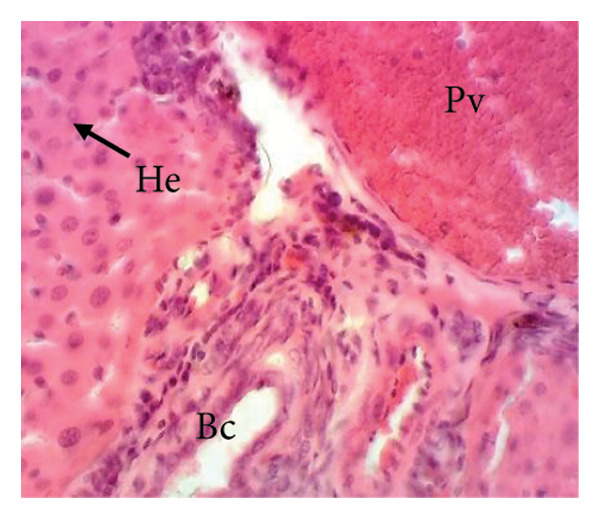
(a)

**Figure:**
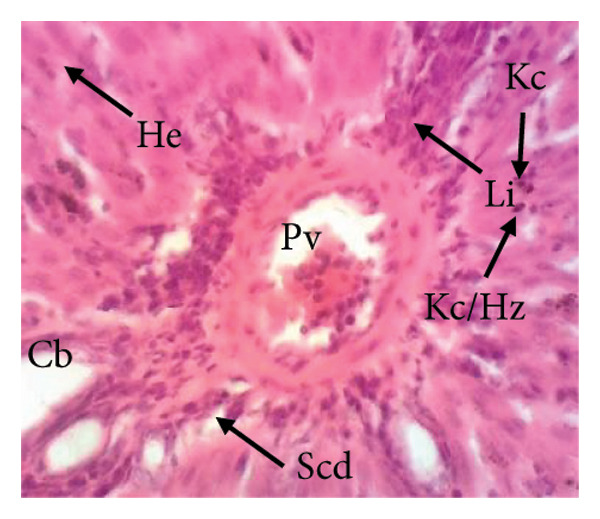
(b)

**Figure:**
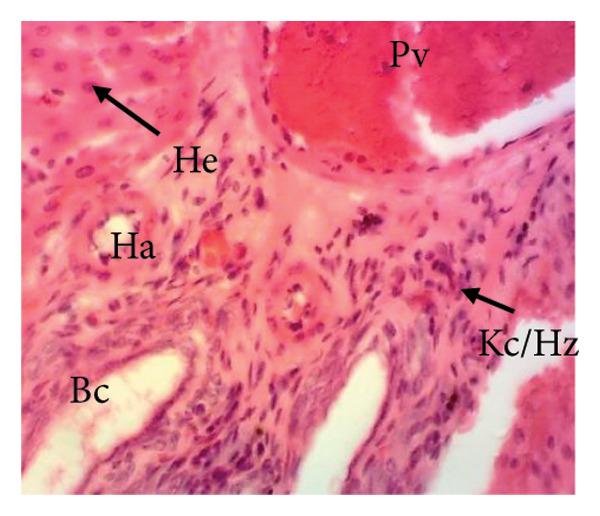
(c)

**Figure:**
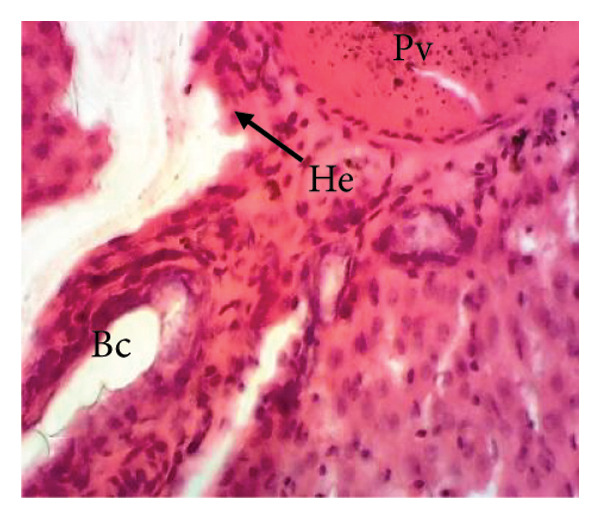
(d)

**Figure:**
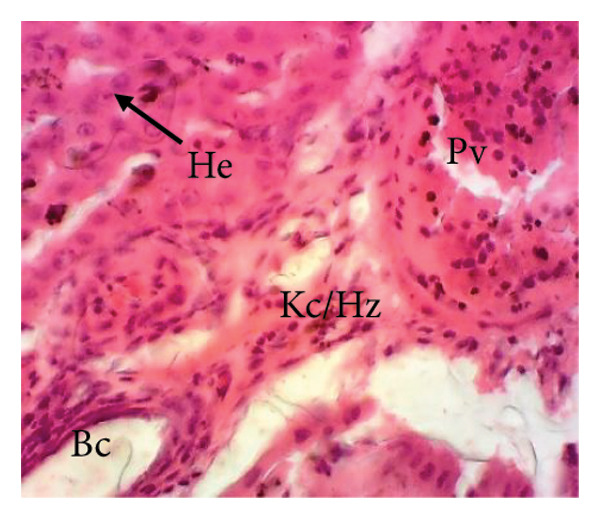
(e)

**Figure:**
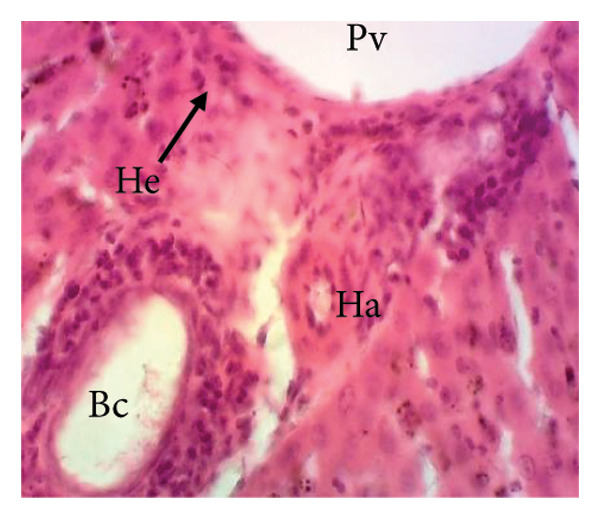
(f)

##### 3.8.2.2. Effects on the Kidneys

Figure 11 depicts the effects of *P. berghei* infection and treatment on kidney tissue. Panel A shows a healthy kidney with a normal appearance: well‐defined glomeruli (G), clear urinary space (US), and distinct distal convoluted tubules (Dct) and proximal convoluted tubules (Pct). In contrast, the kidney of an untreated malaria‐infected animal (Panel B) exhibits alterations in the architecture, including a reduced urinary space and an enlargement of the mesangial area (Em). These changes indicate the potential kidney dysfunction. Treatment with the *E. chlorantha* extract at all administered doses (125, 250, and 500 mg/kg) (Panels D, E, and F) and chloroquine treatment (10 mg/kg) (Panel C) restored the kidney architecture closer to the healthy state observed in Panel A. This suggests that the extract and chloroquine may help protect against kidney damage caused by malaria infection.

Figure 11Effects of *E. chlorantha* stem bark on the microphotography of the kidney section of *Plasmodium berghei*–infected rat (HE × 200). (a) Normal control; (b) malaria control; (c) chloroquine control, (d–f) test groups treated with the respective doses of the extract of 125, 250, and 500 mg/kg. G = glomerulus, Eu = urinary space, Dct = distal convoluted tubes; Pct = proximal convoluted tubes Em = mesangial expansion.

**Figure:**
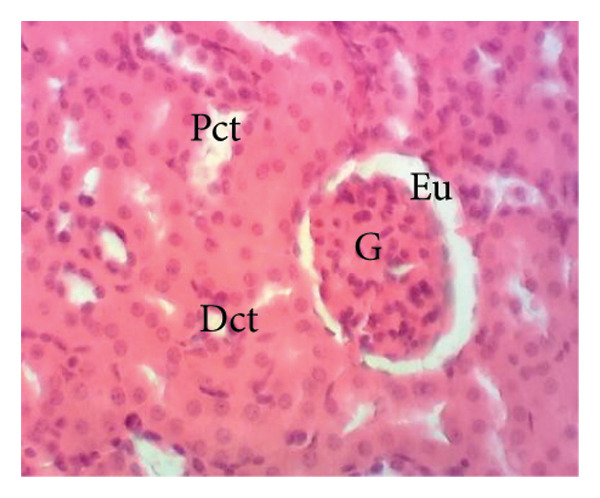
(a)

**Figure:**
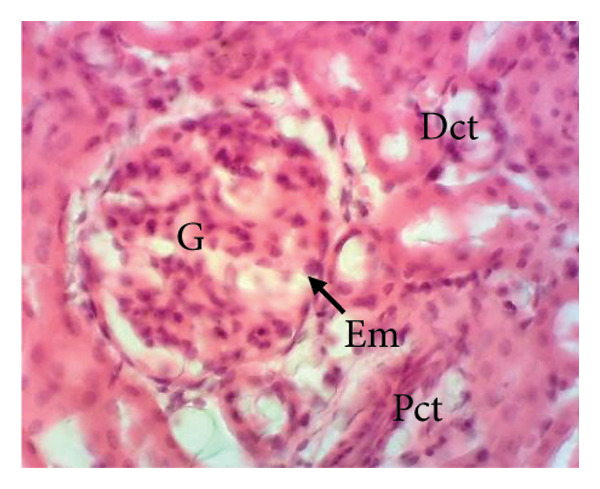
(b)

**Figure:**
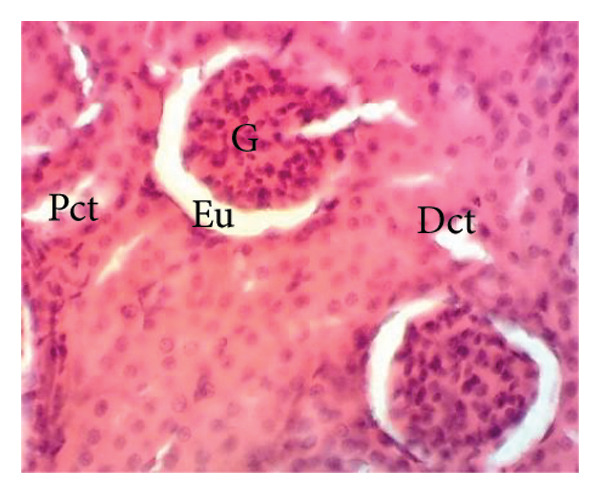
(c)

**Figure:**
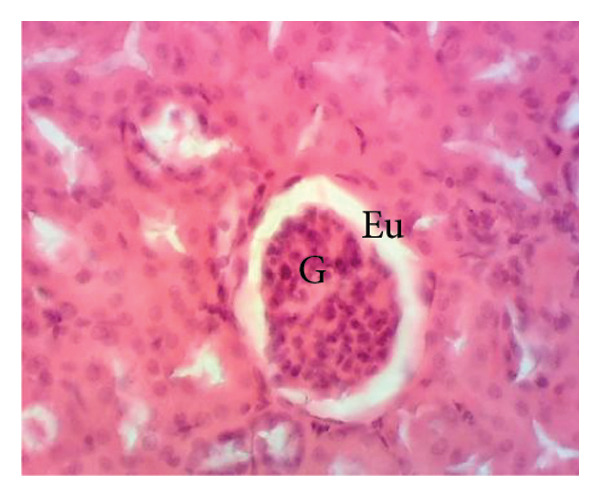
(d)

**Figure:**
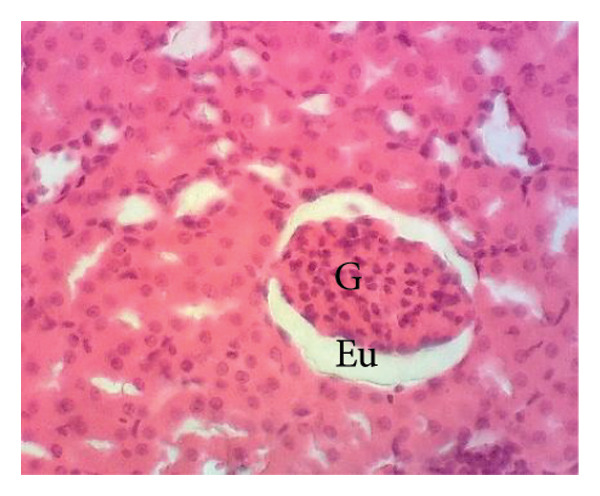
(e)

**Figure:**
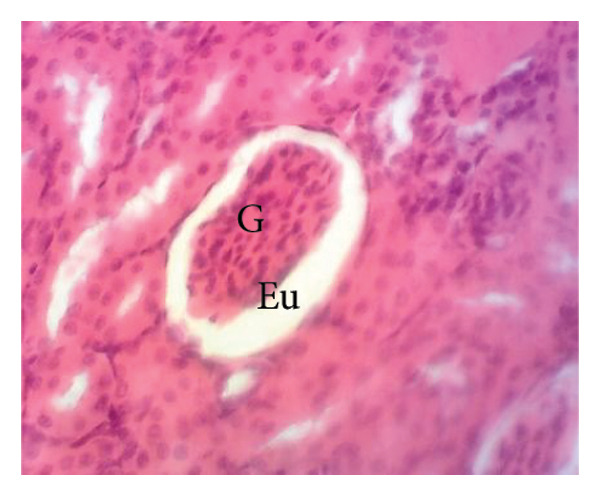
(f)

##### 3.8.2.3. Effects on the Spleen

Figure 12 illustrates the effects of *P. berghei* infection and *E. chlorantha* extract treatment on spleen tissue. Panel A shows a healthy spleen with a well‐organized structure: distinct white pulp (WP), red pulp (RP), and splenic artery (SA). However, the spleen of an untreated malaria‐infected animal (Panel B) presents a disorganized architecture with a loss of clear differentiation between the white and red pulp. This disruption suggests potential functional impairment of the spleen. Treatment with the *E. chlorantha* extract showed an improvement in spleen architecture. At the lowest dose (125 mg/kg), some disorganization remained (Panel D), which disappeared at the higher doses (250 and 500 mg/kg) (Panels E and F) and chloroquine treatment (10 mg/kg) (Panel C) with a restored spleen architecture, closer to the healthy state (Panel A). This suggests that the extract and chloroquine may help preserve normal spleen function during malaria infection.

Figure 12Effects of the *E. chlorantha* extract on the microphotography of the spleen tissue of *Plasmodium berghei*–infected rat (HE × 200). (a) Normal control; (b) malaria control; (c) chloroquine control, (d–f) test groups treated with the extract doses of 125, 250, and 500 mg/kg. Wp = white pulp, Rp = red pulp, Sa = splenic artery, Hz = hemozoin.

**Figure:**
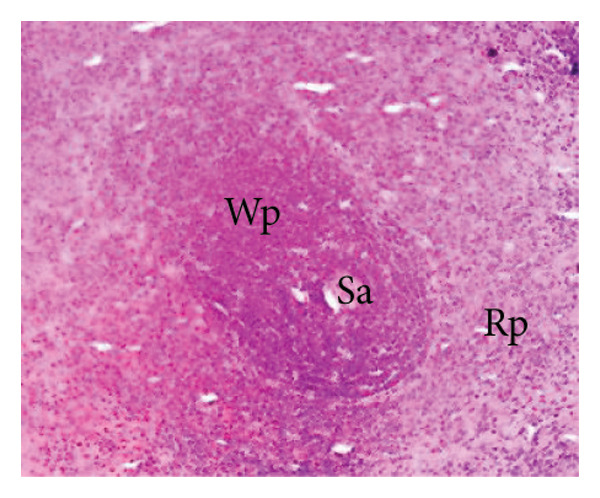
(a)

**Figure:**
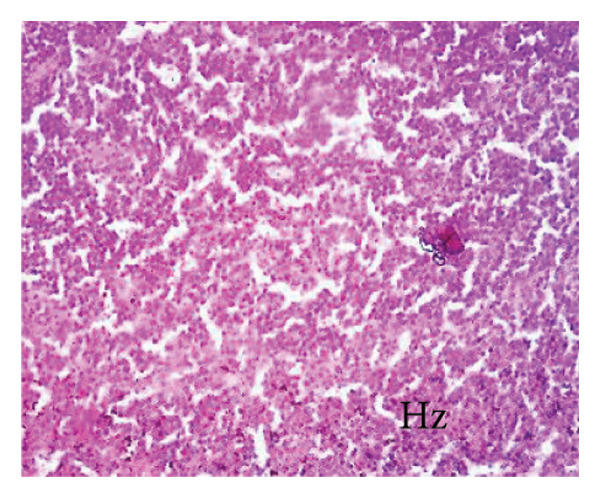
(b)

**Figure:**
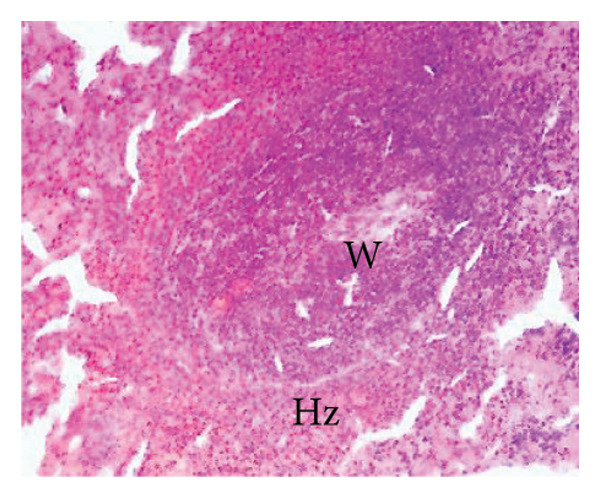
(c)

**Figure:**
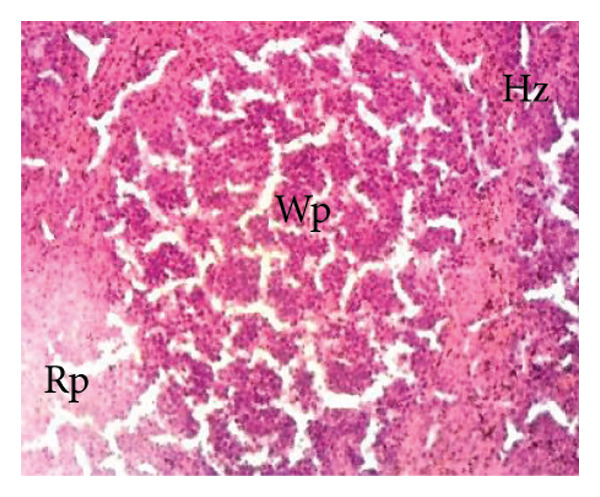
(d)

**Figure:**
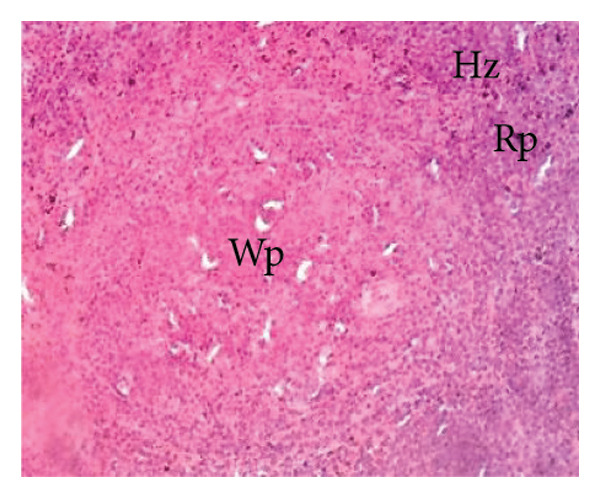
(e)

**Figure:**
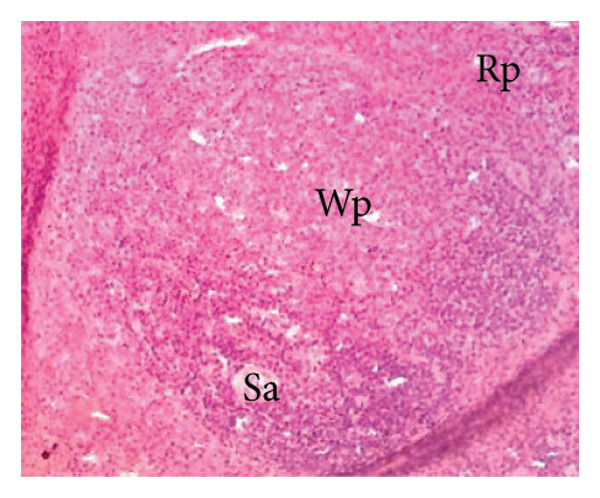
(f)

##### 3.8.2.4. Effects on the Hippocampal Regions and the Dentate Gyrus of the Brain

**Figure 13:**
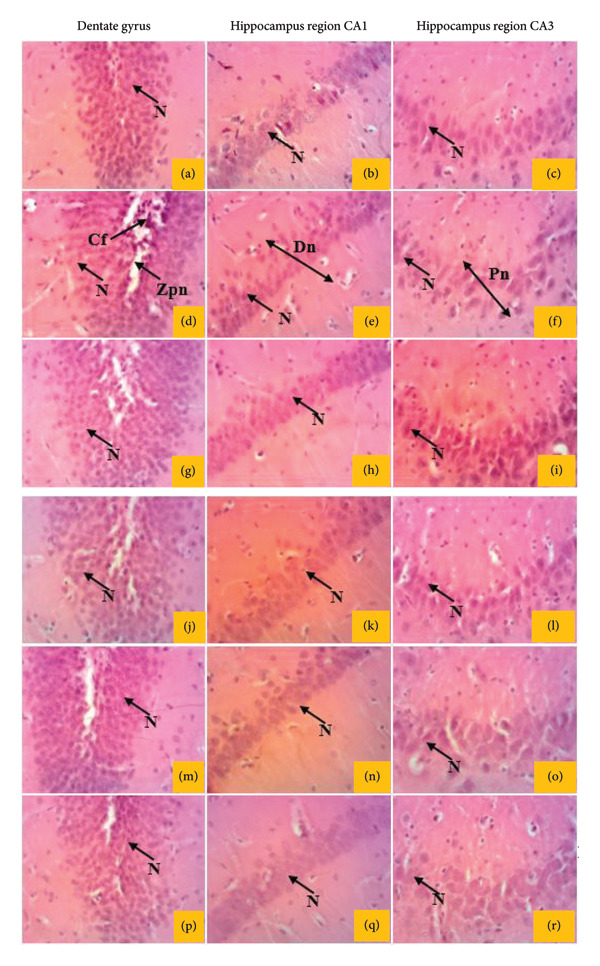
Effects of *E. chlorantha* bark on the microphotography of sections of hippocampal regions CA1 and CA2 and dentate gyrus of the brain tissue of *Plasmodium berghei*–infected rat (HE × 200). (a–c) normal control; (d–f) malaria control; (g–i) chloroquine control, [(j–l); (m–o) and (p–r)] test groups treated with the extract doses of 125, 250, and 500 mg/kg. Nn = normal neuron, Dn = neuronal disruption, Pn = neuronal loss; Zpn = zone of neuronal loss, Cf = fuse cell.

Figure 13 shows the effects of *P. berghei* infection and *E. chlorantha* extract treatment on brain tissue, specifically the dentate gyrus and hippocampal regions CA1 and CA3. Panel (a) (b, c) displays a healthy brain with normal neuronal structure. By contrary, malaria infection (Panels (d–f)) caused severe damage, including extensive neuronal loss in the dentate gyrus (granular cell layer and spindle cells) and a decrease in neuronal density and disorganization within the CA1 and CA3 regions. These alterations suggest potential cognitive impairment. Treatment with the *E. chlorantha* extract at all administered doses (125, 250, and 500 mg/kg) and chloroquine treatment (10 mg/kg) significantly improved the brain structure (Panels (g–l)). This improvement is evident by reduced neuronal loss in the dentate gyrus and a more organized structure in the CA1 and CA3 regions, bringing the overall architecture closer to that of the healthy control group (Panel (a)). These findings suggest the extract and chloroquine may have neuroprotective effects against brain damage caused by malaria infection.

## 4. Discussion

Natural products are widely recognized as a crucial source for new drug discovery, with a long history of traditional use for their significant biological properties across various therapeutic fields [26]. This study investigated the potential of the aqueous extract from *E. chlorantha* stem bark to combat malaria parasites. The extract effectively inhibited the *in vitro* growth of two common *P. falciparum* strains (*PfDd2* and *Pf3D7*), with IC_50_ values of 1.002 and 19.040 μg/mL, respectively. These results indicate the promising antiplasmodial properties. The differential sensitivity observed suggests that the extract maintains its efficacy even against the chloroquine‐resistant PfDd2 strain. This could indicate the presence of compounds that bypass known resistance mechanisms, such as mutations in the *pfcrt* gene, by acting through alternative pathways like disrupting parasite membrane integrity or inhibiting key metabolic enzymes [27]. Further mechanistic studies are essential to pinpoint the exact mode of action and assess its potential to overcome existing drug resistance. Similarly, when administered to infected rats (*in vivo*), the extract significantly reduced parasite burden in a dose‐dependent manner. This confirms the antiplasmodial efficacy of the extract. This activity may be attributed to bioactive compounds like citreoisocoumarin, which is known to inhibit hemozoin polymerization, decrease mitochondrial membrane potential, and inhibit DNA gyrase [28]. Another compound, eucapsitrione, an anthraquinone derivative, has been shown to induce cellular cycle arrest and apoptosis [29]. Additionally, other phytoconstituents such as alkaloids, flavonoids, and saponins may contribute to antiplasmodial activities. Alkaloids can hinder the formation of beta‐hematin (hemozoin/malaria pigment) and interfere with parasite cell division [30], while flavonoids and phenols can disrupt FAS, inhibit the mitochondrial respiratory chain, and alter the parasite’s redox balance [24, 31–33]. The collective action of these constituents could lead to the parasite’s death.

The observed decrease in body weight in untreated infected animals is likely a result of reduced food intake during the malaria infection [34, 35]. In this study, animals treated with the extract gained weight similar to the healthy control group, suggesting that the extract has a protective effect against weight loss associated with malaria [36].

Anemia is a well‐known consequence of malaria, resulting from the parasite’s destruction of RBCs. The aqueous extract of *E. chlorantha* stem bark, rich in flavonoids and phenols, appeared to modulate blood parameters and potentially enhance resistance to erythrocyte hemolysis, thereby helping to prevent parasite‐induced anemia in the mice [37]. Furthermore, the extract significantly protected animals from an increase in the WBC count, highlighting its protective effects against the harmful effects of the infection. Alterations in biochemical parameters such as transaminase activities (ALT and AST), bilirubin, total protein, and creatinine levels are established markers of organ function. Our findings showed that malaria infection led to significant changes in these markers, indicative of potential liver and kidney dysfunction [4]. Treatment with the *E. chlorantha* extract modulated these parameters, suggesting a protective effect on both liver and kidney function. This is likely due to the presence of compounds such as leucopeonidin (flavonoids), eucapsitrione (polyketide), and other phenolic compounds and alkaloids known for their hepatoprotective properties [38, 39]. Indeed, anthraquinones prevent oxidative stress by inhibiting ROS generation and mitochondrial damage through the AMP‐activated protein kinase (AMPK)/Yes‐associated protein (YAP)–mediated pathway in a hepatocyte cell line [40]. Flavonoid inhibits renal inflammatory and cell apoptosis​ [41], suppresses nuclear factor‐κB and mitochondrial apoptosis pathways, and activates the Nuclear Factor Erythroid 2–Related Factor 2/Heme Oxygenase‐1 pathway [42]. The nephroprotective effect of tannins could be attributed to the glycosidation with glucose [43]. Additionally, the observed recovery of these parameters could be a secondary effect of the significantly reduced parasitemia, which lessens the impact of RBC cytoadherence and hemoglobin crystal precipitation in renal tubules [41, 44]. The observed recovery of liver and kidney parameters suggests a potential organ‐protective effect of the extract. However, it is also possible that this recovery is primarily a consequence of reduced parasite burden and overall improvement in the health status of the host. While the data indicate a beneficial effect on organ function, further studies are necessary to determine whether the extract exerts a direct protective action on hepatic and renal tissues or whether the improvements are secondary to its antiparasitic activity. Regarding specific biometabolites, certain phytochemicals identified in the extract—such as flavonoids, phenolic acids, and other antioxidant compounds—are known to possess hepatoprotective and nephroprotective properties. For example, flavonoids like quercetin have been reported to mitigate oxidative stress and inflammation in liver and kidney tissues. Future mechanistic studies focusing on these compounds could elucidate their potential roles in mediating the organ‐protective effects observed. The alterations in serum parameters of infected animals were associated with the onset of oxidative stress, a known adverse effect of malaria. This stress is caused by the host’s inflammatory response, free radical production from hemoglobin breakdown, and direct free radical generation by the parasite [45, 46]. Oxidative stress in this study was evident from decreased levels of antioxidants (catalase, SOD, and GSH) and increased MDA concentration in untreated animals. The observed upregulation of antioxidant enzymes and the downregulation of MDA in treated animals suggest the extract’s ability to enhance antioxidant capacity and reduce lipid peroxidation [47]. This may be due to the extract’s phytoconstituents—including alkaloids, flavonoids, and phenols—which are known to combat reactive oxygen species (ROS) [48]. Alkaloids can manage oxidative stress by activating the PI3‐K/AKT signaling pathway [49], while tannins inhibit the MAPK signaling pathway activated by H_2_O_2_ in parasites, protecting cells [50]. Anthraquinones such as 1‐benzylanthraquinone or eucapsitrione have remarkable abilities in scavenge free radicals and prevent oxidative damage to tissues [51]. It was demonstrated that Citreoisocoumarin exhibits neuroprotective effects by scavenging free radicals and reducing amyloid fibrillization. It has been shown to protect against hydrogen peroxide–induced neurotoxicity in cell culture models [52]. Although the specific relationship between the antioxidant mechanism and the antioxidant activity *in vivo* has not been clarified, some information can be obtained from the bioactivity and pharmacokinetic studies. In general, the mechanism for enhancing the activity of antioxidant enzymes may relate to the redox activity of anthraquinones or their ability to bind specific proteins or both. These antioxidant properties contribute to tissue repair. Likewise, the observed neuroprotective effects, including reduced neuronal loss and a more organized hippocampal structure of the treated animals with the extract, are indeed promising. These could include its known antioxidant properties, which help mitigate oxidative stress—a major contributor to neuronal damage during infection. Additionally, anti‐inflammatory effects may reduce neuroinflammation, further protecting neuronal integrity. While direct antiparasitic effects within the brain cannot be entirely ruled out, it is more likely that the extract’s neuroprotective benefits are mediated primarily through its anti‐inflammatory and antioxidant activities. Further mechanistic studies are required to explore these pathways in greater detail.

## 5. Conclusion

The aqueous extract of *E. chlorantha* stem bark demonstrated moderate‐to‐good *in vitro* antiplasmodial activity against the Dd2 and 3D7 strains of *P. falciparum*. In infected animals, the extract inhibited parasite growth, preventing body weight loss, regulated serum transaminases, bilirubin and creatinine levels, anemia, and leukocytosis. The treatment also protected against oxidative stress and organ damage. These beneficial effects could result from compounds identify in the extract such as citreoisocoumarin, 1‐benzylanthraquinone, leucopeonidin, and eucapsitrione that contribute to destroy the parasite and restore physiological parameters altered by infection. These observations support the traditional use of *E. chlorantha* stem bark in malaria treatment and suggest its antimalarial properties. However, the extract’s exact pharmacological mechanism of action requires further investigation.

**Nomenclature**
ALT: Alanine aminotransferase
AST: Aspartate aminotransferase
CC50: 50% cytotoxic concentration
DMEM: Dulbecco’s modified Eagle medium
DMSO: Dimethylsulfoxide
DNA: Deoxyribonucleic acid
EDTA: Ethylene diamine tetra‐acetic acid
FAS II: Fatty Acid Synthesis Type II
GSH: Reduced glutathione
GSH‐Px: Glutathione peroxidase
Hb: Hemoglobin
HCl: Chlorohydric acid
HCT: Hematocrit
HE: Hematoxylin and eosin
HFF: Human foreskin fibroblast
HNC: National Herbarium of Cameroon
LC/MS: Liquid chromatography–mass spectrometry
MAPK: Mitogen‐activated protein kinases
MDA: Malondialdehyde
NO: Nitric oxide
OD: Optical density
PI3‐K/AKT: Phosphatidylinositol 3‐Kinase/Protein Kinase B
PLT: Platelet
QTOF: Quadrupole time‐of‐flight
RBC: Red blood cell
ROS: Reactive oxygen species
SI: Selectivity index
SOD: Superoxide dismutase
TAE: Tannic acid equivalent
UHPLC‐MS: Ultrahigh performance liquid chromatography‐mass spectrometry
WBC: White blood cell
WHO: World Health Organization

## Conflicts of Interest

The authors declare no conflicts of interest.

## Author Contributions

Abel Narcisse Messi Betene: plant collection, extraction, methodology, investigation, software, writing–original draft, and writing–review and editing. Raceline Gounoue Kamkumo: conceptualization, investigation, data curation, writing–original draft, and writing–review and editing. Patrick Valère Tsouh Fokou: data curation and writing–review and editing. Florence Ngueguim Tsofack: data curation and writing–review and editing. Michel Arnaud Mbock: methodology, phytochemical studies, data curation, software, visualization, and writing–review and editing. Eugenie Aimée Madiesse Kemgne: data curation, writing–review and editing. Albertine Ngako: methodology and writing–original draft. Loic Steve Ngwem Tenlep: phytochemical studies, data curation, and writing–review and editing. Marius Jaures Tsakem Nangap: investigation and methodology. Roberto Fokou: investigation and methodology. Darline Dize: methodology, data curation, and writing–review and editing. Pascal Owona: histological study. Mariscal Brice Tchatat Tali: investigation, methodology, and data curation. Fabrice Fekam Boyom: conceptualization and supervision. Théophile Dimo: conceptualization and supervision.

## Funding

This research did not receive any specific grant from funding agencies in the public, commercial, or not‐for‐profit sectors.

## Data Availability

The data that support the findings of this study are available from the corresponding author upon reasonable request.
